# Parental Burnout and Adolescents’ Development: Family Environment, Academic Performance, and Social Adaptation

**DOI:** 10.3390/ijerph20042774

**Published:** 2023-02-04

**Authors:** Wei Wang, Shen Chen, Shengnan Wang, Geyan Shan, Yongxin Li

**Affiliations:** Institute of Psychology and Behavior, Henan University, Kaifeng 475001, China

**Keywords:** parental burnout, parental psychological control, family environment, academic performance, interpersonal relationship

## Abstract

The present study aimed to examine the effect and underlying mechanism of parental burnout on adolescents’ development as well as the mediating role of parental psychological control. Adolescents’ academic performance, and social distress were selected as developmental indicators. Data were collected on three different occasions using a time-lagged design. Questionnaires were distributed to 565 Chinese families. In the first phase of data collection, fathers and mothers were asked to provide data regarding their parental burnout separately. In the second phase, adolescents were asked to provide details regarding their perceived father and mother’s psychological control. In the third phase, adolescents were asked to provide information on their social distress. At the end of their term, academic performance scores on the final exams were collected. In total, data of 290 students (135 boys; *M*_age_ = 13.85 years) and their parents (for fathers age *M* = 41.91, and for mothers *M* = 40.76) were matched. The results of the multi-group structural equation model showed that parental burnout was negatively related to adolescents’ development indirectly through parental psychological control. Parental psychological control partial mediated the relation between parental burnout and academic performance, and fully mediated the relation between parental burnout with social adaptation. In addition, mothers’ parental burnout showed a stronger effect than fathers’. Mothers’ parental burnout generally showed significant effects on adolescents’ development, while the same indirect effects were not significant in the sample of fathers. These results showed the importance of mothers’ influence on adolescents in parenting activities, and therefore, attention should be paid to mothers in the intervention and prevention of parental burnout.

## 1. Introduction

Having a child may bring great pleasure and new meaning in parents’ lives; however, the experience of parenting can be stressful [1]. If parents are unable to cope well with stress, they may develop parental burnout [2]. Research has indicated that parental burnout may be positively related to addiction behavior, suicidal ideation, escape ideation, and sleep problems. Furthermore, parental burnout may also increase conflict and estrangement toward partners or spouses [3,4].

While parenting activities eventually affect their offspring, the healthy growth of children should also be a criterion of parenting quality. Research has revealed the negative impact of parental burnout on the development of children and adolescents. For instance, parental burnout could increase the risk of neglectful and violent behavior toward their offspring [3,4], worsens the quality of the parent-child relationship [5], may make parents regret having a child [6], and increases depressive and anxiety symptoms in adolescents [7].

These studies have significantly improved our understanding of the consequences of parental burnout. However, previous studies have several limitations. First, they only examined one or two aspects of children and adolescents’ healthy development and few studies examined the effect of parental burnout on children’s and adolescents’ development directly [8]. However, the development of children and adolescents may include several key indicators from different domains, such as the family environment, academic performance, and social adaptation [9]. Second, most extant studies relied on parents’ perspectives and lacked data from different resources. In other words, the developmental indices of the children were reported by their parents. Only a few studies have relied on the answers of the children themselves [7,8]. Thus, it is necessary to supplement data using a multi-informant approach such as children themselves or their schools. Third, previous studies have separately examined the effects of either fathers’ or mothers’ parental burnout on children’s development; few studies consider the family as a whole. However, according to the social role theory [10] and family system theory [11], fathers and mothers may have different gender role expectations, resulting in different levels of involvement in childcare and education. Therefore, through the mother-children or father-children subsystem, the effects and underlying mechanisms of fathers’ and mothers’ parental burnout on children’s healthy development indicators may also be different.

**Figure 1:**
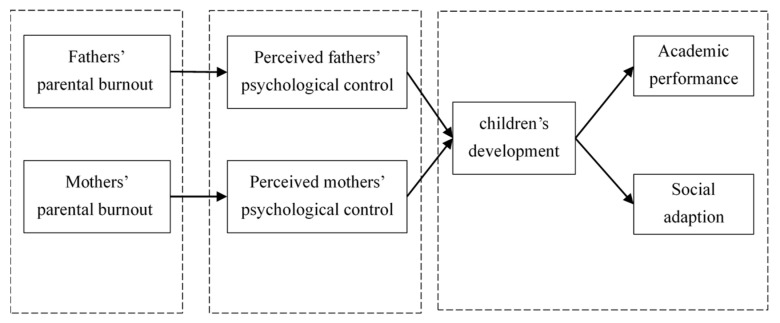
The Framework of the Present Study.

To address this gap, the present study employed a time-lagged design, which assessing independent variables, mediation variable, and dependent variables at three phases. to examine the effect and underlying mechanism of parental burnout on children’s development. Adolescents show rapid physical maturity; however, their psychology cannot develop as quickly as their physical maturity [12]. This may cause them to experience various problems, such as increasing conflict with their parents or other behavioral and emotional concerns [13]. In addition, adolescents’ desire for greater autonomy is significantly related to their mothers’ stress [14]. Therefore, this study focused on a sample of adolescents and their parents. The adolescents’ academic performance (provided by the school), and social adaptation (operationalization as adolescents’ social distress, provided by adolescents themselves) were selected as their health developmental indicator [9]. In addition, the mediating role of adolescents’ perceived parental psychological control was examined. The framework of the present study is shown in Figure 1.

### 1.1. Key Indicators of Adolescent’s Healthy Development

There are various aspects to adolescents’ development. For instance, a large-scale survey including hundreds of researchers and over 100 thousand participating adolescents was conducted in China [9]. The results were published as the “Key Indicators of Psychological Development of Children and Adolescents Aged 6 to 15 in China”, provided a fundamental framework of children and adolescents’ developmental indicators. In their work, they chose indicators from four aspects of adolescents’ development: cognitive ability, academic achievement, social adaptation, and the developmental environment. Their work provided a comprehensive perspective of children and adolescents’ development.

Because learning is one of adolescents’ main work, their academic performance should be an important indicator of adolescents’ development. Therefore, the present study used academic performance as an indicator to assess adolescents’ academic achievement. Further, as a vital indicator of social adaptation, social distress is defined as the experience of negative emotions, such as being distressed, tense, upset, or anxious in social interactions or the reported lack of positive emotion (e.g., being relaxed, calm, at ease, or comfortable). High social distress is related to uncomfortable feelings in social situations [15] and a lower level of self-confidence, need for dominance, need for affiliation, and need for change [16]. The present study used social distress to assess the social adaptation. Based on the framework of prior study [9], the present study chose various variables, including academic performance and social distress, as indicators to assess the effects of parental burnout on adolescents’ development.

### 1.2. Parental Burnout and Adolescents’ Development

Parental burnout refers to a series of negative symptoms that stem from chronic parenting stress and lack of resources [17]. The symptoms include four dimensions: overwhelming emotional exhaustion from the partnering role, contrast with previous self-as-parents, being fed up with the parental role, and emotional distancing from the adolescent [18]. According to the Balance between Risks and Resources (BR^2^) model of parental burnout [17], parental burnout occurs when parenting demands chronically overpower the available resources. The BR^2^ Model was originally proposed to explain the occurrence of parental burnout, it may also provide a framework to explain the consequences of parental burnout. Burned-out parents are already exhausted and lack parenting resources to cope with their children’s needs. According to the conservation of resources (COR) theory [19], individuals seek to protect and promote their resources; perception of resource loss, a threat to resources, and/or inability to gain new resources can result in stress responses. Parents who experience parental burnout tend to protect themselves from further loss of parenting resources. Hence, they stop involving more resources, become emotionally distant, and take care of their children on autopilot [17]. Living with burned-out parents, children cannot get as much care and attention from their parents as in the past. Consequently, parental burnout may be related to undesirable parenting outcomes in children. In line with prior studies [5,8], Hypothesis 1 is proposed.

**Hypothesis 1.**  *Parental burnout is negatively related with adolescents’ health development.*

### 1.3. Parental Psychological Control and Adolescents’ Development

Parental psychological control refers to parenting behaviors that intrude, constrain, invalidate, or manipulate children’s psychological and emotional experiences [20]. It includes a series of behaviors, such as inducing guilt in children, withdrawing love from children, constraining children’s self-expression by asserting authority, manipulating and repressing their children, and keeping the children emotionally dependent on parents [21,22]. Previous studies have shown that parents’ psychological control is associated with children’s undesirable developmental outcomes, such as poor school performance [23], negative emotions [24], social aggression [25,26], and other problematic behaviors, including over- and under-eating behaviors, risky cyber behaviors, and substance use [27]. A review work [28] found a significant relationship between parental psychological control and problem behavior. In line with prior studies, Hypothesis 2 is proposed.

**Hypothesis 2.**  *Parental psychological control is negatively related with adolescents’ development.*

Parental psychological control may not only affect adolescents’ development directly but may also play a mediating role in the relationship between parental burnout and adolescents’ development. As burned-out parents may feel extremely exhausted by their parental role. Subsequently, parents who suffer parental burnout may become less involved in parenting and in their relationship with their children, no longer enjoy being with their children [18]. When interacting with their children, they may have a tough or even violent attitude to make their children behave according to their wish. Existing literature shows that parental psychological control is positively correlated with parental burnout [29], and excessive parenting stress is positively related to parental control attitude [30]. Prior study showed that parental psychological stress plays a mediating role in parental depression and problem behavior [31]. Therefore, Hypotheses 3 and 4 are proposed.

**Hypothesis 3.**  *Parental burnout is positively related with parental psychological control*.

**Hypothesis 4.**  *Parental psychological control mediates the relationship between parental burnout and healthy development in adolescents.*

### 1.4. The Present Study

The present study aimed to examine the specific effect of parental burnout on adolescents’ development in various aspects and the mediation effects of fathers’ and mothers’ parental psychological control in a Chinese sample. Based on the framework of prior study [9], adolescents’ academic performance and social distress were chosen as developmental indicators. While mothers are still regarded as the main force of parenting activities, Confucian culture also emphasized the important and core role of fathers in children’s education since ancient times [32]. The Chinese proverb, “If a son is uneducated, his dad is to blame”, indicates that the father was considered to be mainly responsible for the failure of children’s education. Therefore, this study examined the effects of fathers’ and mothers’ parental burnout on children’s development separately.

## 2. Methods

### 2.1. Sample

The participants were middle school students (from a school located in an urban area in central China) and their parents. Three versions of the questionnaires (for adolescents, fathers, and mothers) were distributed to students during their class. The data were collected on three different occasions. In the first phase of data collection, students were instructed to take the questionnaire home (sealed in an envelope) and give it to their parents. After the parents completed the questionnaire, they were asked to seal it in the reply envelope and hand it back the students, who returned them in school. The second and third phases of data collection were completed by the students in the class. At the end of the term, the teachers provided the students’ academic performance scores. Parents’ parental burnout were collected at the first phase, the perceived psychological control of adolescents were collected at the second phase, and the academic performance, and social distress were collected at the third phase.

All the participants were required to sign an informed consent form. They were informed that the purpose of the survey was to determine family relationships and that participation was voluntary. Further, it would not cause any loss if they did not want to participate in the survey. Participants could withdraw from the study at any point of time. This survey was approved by the research ethics committee of the authors’ academic institution.

A total of 398 fathers and 450 mothers completed the parental burnout questionnaire. In the second and third round, 491 students completed the parental psychological control questionnaire, and 378 students completed the social distress questionnaire, respectively. The data from 290 families were matched, including 135 boys, 154 girls, and one who did not report gender, with an average age of 13.85 years (*SD* = 0.63). Fifty-four of them were from single-child families. The average age of the fathers was 41.91 years (*SD* = 4.19), and that of the mothers was 40.76 (*SD* = 4.16). Regarding fathers’ education, 168 had an education of higher school and lower, 117 were graduates from junior college or had a bachelor’s degree, and 5 had a master’s degree or higher. Among mothers, 187 had an education of higher school or lower, 97 had graduated from junior college or had a bachelor’s degree, and 6 had a master’s degree or higher.

### 2.2. Sample Size Estimation

In order to test whether the sample size of our analysis is sufficient, G*Power 3.1 was used to conduct the post hoc power analysis (with effect size f^2^ = 0.015, α = 0.05). The result showed a power of 0.997, indicating that the sample size is sufficient.

### 2.3. Measures

#### 2.3.1. Parental Burnout

Parental burnout was measured using the Chinese version of the Parental Burnout Assessment (PBA; [33]). This scale was translated from the English version [18] and had satisfactory reliability and validity. It consists of 21 items, and each item is rated using a seven-point Likert scale ranging from 1 (completely inconsistent) to 7 (completely consistent), with a higher score representing higher burnout. An example item includes “I feel as though I have lost my direction as a dad/mum;” Cronbach’s α was 0.94 for fathers’ responses and 0.94 for mothers’ responses.

#### 2.3.2. Parental Psychological Control

Parental psychological control was measured using the tool revised by Wang et al. [34]. It consists of 18 items, and each item is rated using a five-point Likert scale ranging from 1 (not at all true) to 5 (very true), with a higher score indicating greater psychological control. As students were asked to evaluate their perceived parents’ psychological control with respect to both their fathers and mothers, the item was modified using father/mother instead of parents. An example item is as follows: “My father tells me that I should feel guilty when I do not meet his expectations”. Cronbach’s α was 0.93 for perceived fathers’ psychological control and 0.92 for perceived mothers’ psychological control.

#### 2.3.3. Academic Performance

Academic performance was elicited using adolescents’ final exam scores at the end of the term. The grade comprised seven subjects: Chinese, Mathematics, English, Physics, History, Morality and law, and Sports. The teachers provided adolescents’ performance scores. We calculated their total score (with or without sports score), the sum and separate scores of the main subject score (i.e., Chinese, Mathematics, English). The results showed no significant changes in the same mediation model. Therefore, we only reported the results for the total score.

#### 2.3.4. Social Distress

Social distress was measured using the Social Distress subscale of the Social Avoidance and Distress Scale [35]. It consists of 14 items, and each item is rated “yes (count as 1)” or “no (count as 0)”, with a higher score indicating greater social distress. An example includes, “I try to avoid situations that force me to socialize”. Cronbach’s α was 0.75.

#### 2.3.5. Demographic Variables

Parents were asked to provide demographic information including age and education level. The adolescents were asked to provide information regarding their age, gender, and whether they were the only child at home.

### 2.4. Data Analysis

Data were analyzed using SPSS 23.0 and AMOS 23.0. First, Welch’s test was conducted to examine the differences between the participants who dropped out and those who did not. Second, descriptive statistics and correlation analysis were used to preliminarily examine the correlations between parental burnout, psychological control, academic performance, social distress, and emotional flexibility. Third, to work with all the available data, multi-group structural equation modeling (SEM) and bootstrapping were conducted to examine the hypothesized model (non-parametric sampling method was used to resample the data, *n* = 5000; bias-corrected bootstrapping was used to calculate the 95% confidence interval (CI)).

## 3. Results

### 3.1. Preprocessing the Data

The results of Welch’s test showed there were no significant differences between adolescents’ age (*t* = −0.38, *df* = 533.41, *p* = 0.706), gender (*t* = −1.76, *df* = 543.15, *p* = 0.079), whether they were the only child in their respective homes (*t* = 0.14, *df* = 527.91, *p* = 0.887), parents’ age (fathers: *t* = 1.09, *df* = 416.87, *p* = 0.278; mothers: (*t* = −0.49, *df* = 478.02, *p* = 0.624), and parents’ education level (fathers: *t* = 0.40, *df* = 414.11, *p* = 0.691; mothers: *t* = −0.42, *df* = 334.69, *p* = 0.676). These results suggest that removing incomplete questionnaires did not result in a bias.

### 3.2. Descriptive Statistics and Correlation Analysis

The descriptive statistics and correlation matrix are presented in Table 1. The demographic variables generally showed no significant correlations with parental burnout (*r*s = −0.07–0.08, n.s.), except for mothers’ age, which showed a significant correlation with mothers’ parental burnout (*r* = 0.13, *p* < 0.05). Mothers’ parental burnout was negatively correlated with adolescents’ academic performance (*r* = −0.13, *p* < 0.05). However, mothers’ parental burnout was not significantly correlated with adolescents’ social distress (*r*s = −0.02, n.s.). Mothers’ parental burnout was correlated with their psychological control (*r* = 0.16, *p* < 0.01), and psychological control was correlated with adolescents’ academic performance (*r* = −0.17, *p* < 0.01), and social distress (*r* = −0.12, *p* < 0.05). Fathers’ parental burnout generally showed no significant correlation with adolescents’ academic performance, social distress (*r*s = −0.06 –0.04, n.s.), or fathers’ psychological control (*r* = −0.06, n.s.). Fathers’ psychological control was correlated with adolescents’ academic performance (*r* = −0.18, *p* < 0.01), and social distress (*r* = −0.12, *p* < 0.05). The indicators showed weak or non-significant correlation with each other (*r*s = 0.01~0.20).

### 3.3. Multi-Group SEM

**Figure 2:**
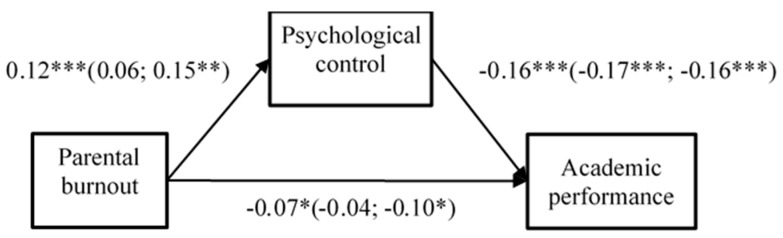
The Results of multi-group SEM (with academic performance as dependent variable). Note. The number outside the brackets is the result of the total sample (*n* = 674), and inside the brackets are the results of fathers (*n* = 317) and mothers (*n* = 357). * *p* < 0.05; ** *p* < 0.01; *** *p* < 0.001.

**Figure 3:**
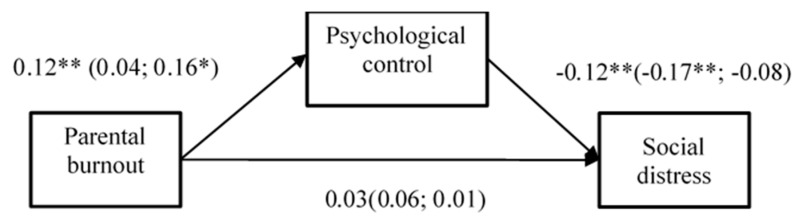
The Results of multi-group SEM (with social distress as dependent variable). Note. The number outside the brackets is the result of the total sample (*n* = 501), and inside the brackets are the results of fathers (*n* = 233) and mothers (*n* = 268). * *p* < 0.05; ** *p* < 0.01.

Because there were still some missing data in each questionnaire, to make full use of our data, a series of multi-group SEM was conducted. Parental burnout was set as an independent variable, parental psychological control as the mediating variable, and the indicators of adolescents’ development were set as the dependent variables. In addition, the unconstrained model, constrained regression weights model, constrained intercepts model, constrained means model, constrained covariances model, and constrained residuals (fully constrained) model were calculated. The models were examined in three groups, that is, the total sample, sample of fathers, and sample of mothers. Furthermore, the Bayesian imputation method was used to examine whether removing incomplete questionnaires could bias our results. The results of multi-group SEM are shown in Figure 2 and Figure 3.

When setting academic performance as the dependent variable, the fully constrained model (equivalent for residuals) showed no difference with the unconstrained model (Δχ^2^ = 18.07, Δ*df* = 18, *p* = 0.451), indicating that groups are not different at the model level; however, they may be different at the path level. In general, parental burnout was positively related to psychological control (β = 0.12, *p* < 0.001) and negatively related to adolescent’s academic performance (β = −0.07, *p* < 0.05), while psychological control was negatively related to academic performance (β = −0.16, *p* < 0.001). The relationship between parental burnout and academic performance was partially mediated by psychological control, the indirect effect was significant (indirect effect = −2.54, SE = 0.84, 95%CI [−4.19, −0.89]). Meanwhile, mothers’ parental burnout was positively related to psychological control (β = 0.15, *p* < 0.001) and negatively related to adolescent’s academic performance (β = −0.10, *p* < 0.05). Moreover, psychological control was negatively related to academic performance (β = −0.16, *p* < 0.001). The indirect effect was significant (indirect effect = −2.77, SE = 1.32, 95%CI [−5.35, −0.18]). However, fathers’ parental burnout was not significantly related to psychological control (β = 0.06, *p* = 0.302) or academic performance (β = −0.04, *p* = 0.397). The indirect effect was insignificant (indirect effect = −1.40, SE = 1.49, 95%CI [−4.32, 1.53]).

When setting social distress as the dependent variable, the fully constrained model (equivalent for residuals) showed significant differences with the unconstrained model (Δχ2 = 41.97, Δdf = 18, *p* = 0.001), indicating that groups are different at the model level. In general, parental burnout was positively related to psychological control (β = 0.12, *p* < 0.001), and psychological control was negatively related to social distress (β = −0.12, *p* < 0.001). The relationship between parental burnout and social distress was fully mediated by psychological control, the indirect effect was significant (indirect effect = −0.06, SE = 0.04, 95%CI [−0.16, −0.01]). However, the mediation effect of psychological control was not significant in the separate sample of fathers or mothers.

The Bayesian imputation method was used to create a complete dataset (five times the original dataset). The goodness of model fit, the regression weights, and the mediation effects of the removed dataset and imputed dataset did not change meaningfully. These results suggest that removing incomplete questionnaires did not significantly bias our results.

### 3.4. Supplementary Analysis

Because academic performance and social distress could also be viewed as antecedents of parental burnout, we have conducted the supplementary analysis to examine these relations. The academic performance and social distress were set as independent variables, psychological control as mediation variable, parental burnout as dependent variable. In line with the results shown in Figure 2 and Figure 3, the results showed that academic performance and social distress could also indirectly affect parental burnout through psychological control.

## 4. Discussion

In recent years, the theme of parental burnout has attracted considerable attention [36]. The present study examined the effect of parental burnout on adolescents’ development from various indicators. Specifically, academic performance, and social distress were chosen as adolescents’ development indicators. As the parents seek to protect and promote their resources, burned-out parents tend to protect themselves from further loss of parenting resources, they may stop to invest more resources to intrude, constrain, invalidate, or manipulate children’s psychological and emotional experiences, result in negative effect on adolescents’ development. We have measured parental burnout, psychological control, and adolescents’ development indicators at three phases, and examined the effects of parental burnout on adolescents’ development, as well as the mediation effects of psychological control. The results generally supported our hypotheses.

### 4.1. Effect of Parental Burnout on Adolescents’ Development

In general, parental burnout showed negative indirect effects on adolescents’ development through parental psychological control, and mothers’ parental burnout showed stronger effects than fathers.

First, parental burnout is negatively associated with adolescents’ academic performance. Burned-out parents may increase their neglect toward the adolescents and stop involving more parenting resources. This may reduce parenting involvement in adolescents’ studies, such as participating in voluntary school activities, communicating and interacting with teachers, participating in family learning activities, or participating in school decision-making and community cooperation [37]. When adolescents have problems with their learning, they may feel less supported and find it hard to ask their parents for help, resulting in a drop in grades. Notably, parental burnout not only affects adolescents’ learning performance but also their sports performance. This suggests that parental burnout may also have a negative impact on the physical development of adolescents. A longitudinal study may need to further examine this relation.

Second, parental burnout has a negative relationship with adolescents’ social distress. Adolescents cannot receive sufficient emotional support from their burned-out parents, resulting in poor parent-child relationships. They were unable to learn effective coping styles and emotion regulation methods when faced with difficulties. Prior studies have also shown that parental burnout is positively related to adolescents’ internalized and externalized problem behaviors [8]. Therefore, our results provide new evidence that parental burnout may worsen adolescents’ social adaptation in terms of social distress.

### 4.2. Effect of Psychological Control on Adolescents’ Development

Both fathers’ and mothers’ psychological control had negative effects on adolescent development. Prior studies have shown that parents may use psychological control to convey their excessive expectations for academic achievement [38]. Experiencing higher levels of psychological control is related to lower feelings of competence [39]. In addition, prior study indicated that Chinese parents tend to consider psychological control as an effective socialization tool to motivate their offspring to meet societal standards [40]. Therefore, when perceived as having a high level of parental psychological control, adolescents may view parents’ behavior in a more manipulative manner and show more rebellious behavior. This results in poor academic performance, and social distress. In line with prior studies with a mixed sample of fathers and mothers, parental psychological control showed a robust effect on adolescents’ development. Owing to the potential increase in adolescents’ need for autonomy and independence, parents’ psychological control may have negative effects on their development in various aspects. Furthermore, among fathers, parental psychological control showed a consistently negative effect on adolescents’ development. This may suggest the traditional concept of “a kind mother and a severe father” being deeply rooted among the Chinese people. Fathers are symbolized as authority and strictness and they have a stronger influence on adolescent development than mothers.

### 4.3. Theoretical and Practical Implications

The present study expands the application of the BR^2^ model [19], which was initially proposed to explain the emergence of parental burnout. This could also provide an explanatory framework for the consequences of parental burnout. Focusing on parenting resources and demands, burned-out parents may result in undesirable consequences for adolescents. In addition, the present study also provided support for the family system theory [11] in the parental burnout literature. As a dynamic system, the exhaustion of parents’ resources will not only increase conflicts with the co-parent but also lead to insufficient investment in parenting adolescents, resulting in family dysfunction.

Furthermore, for adolescents’ development, mothers’ parental burnout generally showed a stronger effect than fathers. Since mothers are still viewed as and remain childcare specialists [41], they may be more involved in childcare events than fathers. Further, researchers have indicated that Chinese mothers play an essential role in providing a secure and loving environment for their adolescents [42]. In the practical work of intervention and prevention of parental burnout, mothers should still be considered the main subject. For instance, existing studies have shown that social support, partner’s co-parenting could decrease the level of parental burnout, therefore, more attention and support needs to be paid to mothers. Meanwhile, as the consistently high relation between father’s parental burnout and psychological control, social support for fathers is also important.

### 4.4. Limitations and Future Directions

Although our findings may improve our understanding of the effects of parental burnout on adolescent development, some limitations still need to be addressed. First, the sample of the present study comprised parents of middle school students. Although parents of middle school students are ideal samples for studying parental burnout, parental burnout may not occur only among adolescents’ parents. Therefore, future studies should collect data from a broader sample, including parents of kindergarten to college students of different ages. Second, the present study adopted a time-lagged design instead of a multi-wave design. Even the time-lagged design could provide better evidence than collecting all data at one time-point, in essence, this study is still a cross-sectional study, which limited us to control the same variable from the first phrase, and unable to make causal inference. Future studies should adopt a more rigorous design to collect all the variables in each period to estimate the robustness of results of the present study. Third, the response rate was lower than expected. We compared the demographic information of the missing data with the retained data, imputed the missing data, and compared the results. Future research needs a more rigorous design to ensure a larger sample and reduce the occurrence of missing values. Fourth, the present study mainly focused on the effects of parents’ parental burnout on the development of their adolescents. However, the reverse effects should not be ignored. Psychological control may be an antecedent of parental burnout. In addition, adolescents’ academic performance and social distress may also have an impact on parents’ burnout and the relation may be bidirectional, for instance, parents with a child presenting learning difficulties or laziness can be much more stressed, therefore, the low academic performance may also be an antecedent of parental burnout. In addition, adolescents’ social distress may result in a series of problematic behaviors, which could increase the risk of parental burnout. Even we have attempt to examine the effects of academic performance, and social distress on parental burnout, temporal sequence of data collecting was not consistent with the logical sequence. Future studies may further consider this reverse relation and examine whether and how adolescents affect parents’ parental burnout.

## 5. Conclusions

Because prior studies about the consequences of parental burnout mainly focused solely on father or mother, and they have focused on the one or two aspects of children and adolescents’ development. To address these gaps, the present study based the BR^2^ theory, examined the effects of parental burnout on adolescents’ health, as well as the mediation effects of perceived parents’ psychological control by using a multi-source data. The results showed that parental burnout was negatively related to adolescents’ development indirectly through parental psychological control. In addition, mothers’ parental burnout showed a stronger effect than fathers’.

## Figures and Tables

**Table 1:** The descriptive statistics and correlation matrix of each variable.

		*M*	*SD*	①	②	③	④	⑤	⑥	⑦	⑧	⑨	⑪	⑫
①	Adolescents’ gender	1.51	0.50	1										
②	Adolescents’ age	13.83	0.81	−0.05	1									
③	Only child	1.25	0.45	−0.08	−0.09 *	1								
④	Fathers’ age	41.92	4.39	−0.02	0.02	0.10	1							
⑤	Mothers’ age	40.84	4.13	0.02	0.01	0.05	0.84 **	1						
⑥	Fathers’ parental burnout	1.75	0.82	−0.06	0.02	−0.02	−0.01	0.02	1					
⑦	Mothers’ parental burnout	1.94	0.91	−0.07	0.05	−0.05	0.08	0.13 *	0.44 **	1				
⑧	Fathers’ psychological control	2.81	0.85	−0.17 **	0.07	0.03	−0.01	−0.02	0.06	0.14 **	1			
⑨	Mothers’ psychological control	2.93	0.85	−0.15 **	0.07	0.03	−0.03	−0.01	0.08	0.16 **	0.87 **	1		
⑩	Students’ academic performance	376.53	104.63	0.20 **	−0.08	0.08	0.05	0.06	−0.06	−0.13 *	−0.18 **	−0.17 **	1	
⑪	Students’ social distress	1.52	0.24	−0.13 *	0.09	0.02	−0.06	−0.02	0.04	−0.02	−0.12 *	−0.12 *	−0.01	1

* *p* < 0.05; ** *p* < 0.01; Adolescents’ gender 1 = boy, 2 = girl.

## Data Availability

The datasets generated for this study are available on request to the corresponding author.
